# A capsule network-based method for identifying transcription factors

**DOI:** 10.3389/fmicb.2022.1048478

**Published:** 2022-12-06

**Authors:** Peijie Zheng, Yue Qi, Xueyong Li, Yuewu Liu, Yuhua Yao, Guohua Huang

**Affiliations:** ^1^School of Electrical Engineering, Shaoyang University, Shaoyang, China; ^2^College of Information and Intelligence, Hunan Agricultural University, Changsha, China; ^3^School of Mathematics and Statistics, Hainan Normal University, Haikou, China

**Keywords:** transcription factors, capsule network, deep learning, LSTM, semantics

## Abstract

Transcription factors (TFs) are typical regulators for gene expression and play versatile roles in cellular processes. Since it is time-consuming, costly, and labor-intensive to detect it by using physical methods, it is desired to develop a computational method to detect TFs. Here, we presented a capsule network-based method for identifying TFs. This method is an end-to-end deep learning method, consisting mainly of an embedding layer, bidirectional long short-term memory (LSTM) layer, capsule network layer, and three fully connected layers. The presented method obtained an accuracy of 0.8820, being superior to the state-of-the-art methods. These empirical experiments showed that the inclusion of the capsule network promoted great performances and that the capsule network-based representation was superior to the property-based representation for distinguishing between TFs and non-TFs. We also implemented the presented method into a user-friendly web server, which is freely available at http://www.biolscience.cn/Capsule_TF/ for all scientific researchers.

## Introduction

Transcription factors (TFs) are also sequence-specific DNA-binding factors, a family of proteins that control the expression of target genes (Karin, 1990; Latchman, 1997). The TFs are widely distributed, and their numbers vary with the size of the genome (Nimwegen, 2006). The larger genomes are likely to have a larger number of TFs on average. Approximately 10% of genes in the human genome are conservatively estimated to code for TFs. Consequently, the TFs are the potentially largest family of proteins in humans. The TFs exert regulating roles alone or together with other proteins in a complex by hindering or facilitating the recruitment of RNA polymerase (a type of enzyme) to specific DNA regions (Roeder, 1996; Nikolov and Burley, 1997). The regulation roles of the TFs are either positive or negative. The TFs promote the recruitment of RNA polymerase function as activators and contrarily ones to hold back recruitment as repressors. The TFs are involved in many important cellular processes including transcription regulation. Some TFs are responsible for cell differentiation (Wheaton et al., 1996), some respond to intercellular signals (Pawson, 1993), and some reply to environmental changes (Shamovsky and Nudler, 2008). Mutations in the TFs are discovered to be implied in many diseases (Bushweller, 2019). The TFs are a control switch to turn on or off to ensure when, where, and how many genes are accurately expressed. Thus, it is a fundamental problem but a therapeutic opportunity for drug discovery and development to accurately identify TFs. Physical or chemical methods (called wet experiments) are a prime alternative to identify TFs. The wet experiments include SELEX-based methods (Roulet et al., 2002), MITOMI (Rockel et al., 2012), and ChIP-based assays (Yashiro et al., 2016). Most known TFs were discovered by wet experiments and deposited in public databases (Wingender et al., 1996; Riaño-Pachón et al., 2007; Zhu et al., 2007; Zhang et al., 2020). The wet experiments accumulated a limited number of TFs at the expense of an enormous amount of time and money. It is only by the wet experiments that it is impossible and insufficient to discover all TFs in all the tissues or species all over the world. With advances in artificial intelligence, it is becoming possible to learn a computational model from these known TFs to recognize new unknown TFs which will be subsequently examined by the wet experiments. The computational methods shrank greatly the numbers of potential TFs that the wet experiments scanned, and thus, save a vast volume of time and money. The computational methods are becoming essentially complementary to the wet experiments, and both are jointly accelerating the exploration of the TFs.

To the best of our knowledge, Liu et al. (2020) pioneered the first computational method for discriminating TFs from non-TFs. Liu et al. extracted three types of sequence features: composition/transition/distribution (CTD) (Tan et al., 2019), split amino acid composition (SAAC), and dipeptide composition (DC) (Ding and Li, 2015). Comprehensively comparing the contribution of features and performances of five frequently used machine learning algorithms: logistic regression, random forest, k-nearest neighbor, XGBoost, and support vector machine (SVM). Liu et al. finally chose 201 optimal features and SVM for building the classifier. Liu et al. opened an avenue to identify TFs. Lately, Li et al. (2022) created a different idea from Liu et al. to distinguish TFs and non-TFs. Instead of designing sophisticated features. Li et al. directly took the sequence as input, split three amino acid residues as a basic unit, and employed long short-term memory (LSTM) for capturing semantic differences between TFs and non-TFs. Li et al. promoted the predictive accuracy to 86.83%. The LSTM is a special recurrent neural network (RNN) which suffered from the long-distance dependency. The capsule network proposed is a novel neural work architecture (Sabour et al., 2017), whose remarkable advantage is to capture relationship between local parts. This just made up for the deficiency of LSTM. Inspired by this, we proposed a capsule network-based method for TFs prediction.

## Materials and methods

### Data

The training and the testing data were downloaded from the website^1^ (Li et al., 2022), which was manually collected by Liu et al. (2020). The original dataset contained 601 human and 129 mouse TFs which preferred methylated DNA (Graves and Schmidhuber, 2005; Wang et al., 2018) and 286 TFs which preferred non-methylated DNA (Yin et al., 2017). Liu et al. (2020) conducted the following steps for improving the quality of the dataset. The sequences containing illegal characters such as “X”, “B”, and “Z” were first removed. Then, the CD-HIT, which is a clustering tool (Huang et al., 2010; Zou et al., 2020), was used to decrease redundancy between sequences. The cutoff threshold was set to 0.25, meaning that the sequence identity between any two sequences was no more than 0.25. Third, less than 50 amino acid sequences were excluded. A total of 522 TFs were finally preserved as positive samples after the above three processes. Liu et al. sampled the same number of non-TFs from the UniProt database (release 2019_11) which meets the following five requirements: (1) reviewed proteins, (2) proteins with evidence at protein level, (3) proteins in full length and of more than 50 amino acid residues, (4) proteins without DNA-binding TF activities, and (5) Homo sapiens proteins with less than 25% sequence identity in the CD-HIT. Liu et al. divided the data further into the training and the independent test dataset at the ratio of 8:2, with the former containing 406 positive and 406 negative samples, and the latter containing 106 positive and 106 negative samples.

### Methods

**FIGURE 1:**
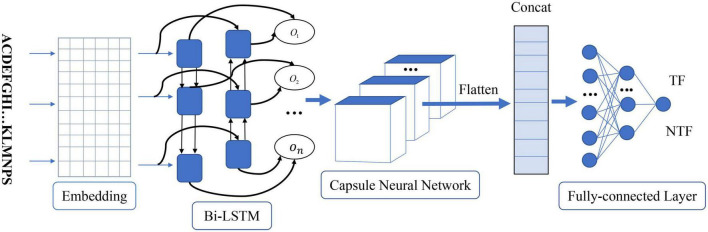
The architecture of the proposed capsule network-based method. *O_n_* represents the word vector after the bidirectional LSTM layer. TF and NTF represent the final predicted outcome as a transcription factor or not.

As shown in Figure 1, the proposed method called Capsule_TF is a deep learning-based method. It mainly contains five layers, namely, embedding layer, bidirectional LSTM layer, capsule network layer, and three fully connected layers. The protein sequence as input goes through the embedding layer and is then embedded into low-dimensional vectors. The bidirectional LSTM layer and the capsule network layer are used to extract high-level representations of protein sequences. Three fully connected layers are finally used to discriminate TFs from non-TFs. The Capsule_TF is an end-to-end deep learning model without designing any features.

#### Embedding

It is mandatory for text sequence input to be converted into digital sequences which are suitable to be processed by the subsequent machine learning algorithms. There are many ways of converting text sequences into digital sequences, such as a one-hot encoding scheme (Buckman et al., 2018) and Word2vec (Rong, 2014). The one-hot encoding scheme fails to capture relationships between words and is opt to yield sparse representation when the vocabulary is large. It is a common practice to use embedding to translate text sequences into dense digital vectors. In the field of text analysis by the deep neural network, the embedding is generally the first layer generally defined by


(1) $${\hat{x}}_{i}=W_{e}x_{i}$$

where ${\hat{x}}_{i}$ denotes the embedding of the word, *x_i_* represents input, and *W*_*e*_ ∈ *R*^*n*×*k*^ denotes a lookup table that stores the embedding of words. *W_e_* is the learnable parameter.

#### Long short-term memory

**FIGURE 2:**
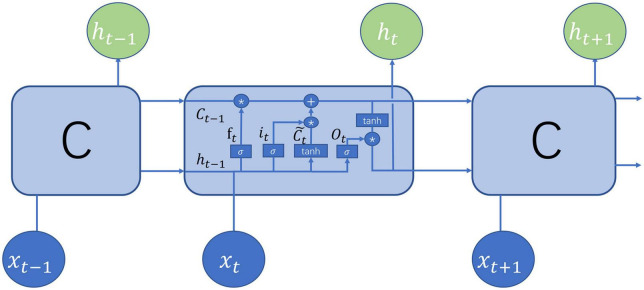
Illustration of long short-term memory (LSTM) structure (Olah, 2015).

The LSTM (Hochreiter and Schmidhuber, 1997) belongs to the family of recurrent neural networks (RNNs) (Sherstinsky, 2020), which is typically a neural network sharing parameters at all time steps. The LSTM was pioneered by Hochreiter and Schmidhuber (Hochreiter and Schmidhuber, 1997) and later was continuously improved. The structure of the current LSTM was mainly made up of the cell state, the hidden state, the input, and the output. Figure 2 demonstrates the structure of the LSTM at the time step t which is identical at all the time steps. The cell state preserved memories for preceding words but was regulated by the gates to determine how much information was conveyed to the next time step. There are three gates in the LSTM: forget gate, input gate, and output gate. The forget gate is defined as


(2) $$f_{t}=\sigma \,\left(W_{f}\cdot \left[h_{t-1},x_{t}\right]+b_{f}\right)$$

where *h*_*t–1*_ denotes the hidden state at time step *t-1*, *x_t_* is the input at time step t, *W_f_* and *b_f_* are learnable parameters, and σ is the sigmoid function. Obviously, the output of the forget gate falls between 0 and 1. The input gate and the candidate cell are defined, respectively, as


(3) $$i_{t}=\sigma \,\left(W_{i}\cdot \left[h_{t-1},x_{t}\right]+b_{i}\right)$$

and


(4) $${\tilde{C}}_{t}=t\,a\,n\,h\,\left(W_{c}\cdot \left[h_{t-1},x_{t}\right]+b_{c}\right)$$

where *W_i_*, *W_c_*, *b_i_*, and *b_c_* are learnable parameters. The cell state is updated by


(5) $$C_{t}=f_{t}*C_{t-1}+i_{t}*{\tilde{C}}_{t}$$

The preceding information is all forgotten if the forget gate is 0, namely, *f*_*t*_ = 0, all the information is born in mind if *f*_*t*_ = 1, and part are born if *f_t_* is more than 0 but less than 1. Obviously, the forget gate determines how much memories for preceding words are preserved. The input gate and the candidate cell determine how much new information about the time step is added to the cell state. The contribution of the time step t to the cell state is nearly nothing if the second item in Equation (5) is equal to 0. The hidden states are updated jointly by the cell state and the output gate


(6) $$h_{t}=O_{t}*\tanh\left(C_{t}\right)$$

where *O_t_* denotes the output gate which is computed by


(7) $$O_{t}=\sigma \,\left(W_{o}\,\left[h_{t-1},x_{t}\right]+b_{o}\right)$$

Compared with the traditional RNN, the LSTM solved well long-term dependency issues by the cell state conveying memory. To capture both directional dependencies between words, the bidirectional LSTM was used here. Due to its efficiency and effectiveness in sequence analysis, the LSTM has been widely applied to the N6-methyladenosine prediction (Chen et al., 2022), speech recognition (Sak et al., 2015), continuous B-cell epitope prediction (Saha and Raghava, 2006), N4-Acetylcytidine prediction (Zhang et al., 2022), lysine succinylation identification (Huang et al., 2021), sentiment analysis (Arras et al., 2017), and action recognition (Du et al., 2015).

#### Capsule network

**FIGURE 3:**
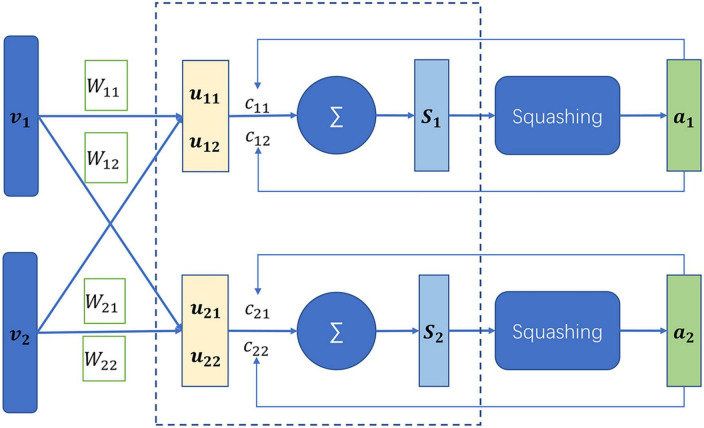
Illustration of dynamic routing in the capsule network (Lee, 2018).

The capsule network is a newly developed neural network in 2017 (Sabour et al., 2017). The capsule network is different from the conventional neural network. The basic unit of the capsule network is capsules which are defined as a set of neurons, while the latter consists of neurons. The neuron is generally a scalar value that represents a single pattern, while the capsules are a multi-dimensional vector, being able to represent multi-patterns. In addition, the capsule network is capable of capturing links between different local properties (Jia and Meng, 2016; Xi et al., 2017), which the convolution neural network (Shin et al., 2016) fail to discover. At the heart of the capsule network lies the dynamic routing as illustrated in Figure 3. *v_i_* was assumed to be the capsules in the layer L, whose prediction vectors are defined by


(8) $$u_{j|i}=W_{ij}v_{i}$$

where *W*_*ij*_ is a learnable matrix. The capsule *s_j_* in the layer L+1 denotes a weighted sum over the prediction vectors, which is computed by


(9) $$s_{j}=\sum_{i=1}c_{ij}u_{j|i}$$

where *c*_*ij*_ is the coupling coefficient. The output of the capsule *s_j_* is further activated by a non-linear “squashing” function so that short vectors get shrunk to almost zero length and long vectors get shrunk to a length slightly below 1.


(10) $$a_{j}=\frac{||s_{i}||}{1+{||s_{i}||}^{2}}\,\frac{s_{i}}{||s_{i}||}$$

The coupling coefficient represents the probability of two capsules to the couple. The more consistent the two capsules, the large the coupling coefficient. The coupling coefficient is initialized as the log prior probabilities that the capsule *j* was coupled to the capsule *i*.


(11) $$c_{i\,j}=\frac{\exp\left(b_{i\,j}\right)}{\sum_{k}\exp\left(b_{k\,j}\right)}$$

The prior probabilities are updated by the dynamic routing algorithm


(12) $$b_{ij}=b_{ij}+a_{j}u_{j|i}$$

The dynamic routing algorithm is to iterate the Equations (9) to (12).


(13) $$u_{i}=W_{i}\,v_{i}$$

## Metrics

For binary classification, there are four common metrics: sensitivity (Sn), specificity (Sp), accuracy (Acc), and Matthews correlation coefficient (MCC), which are defined by


(14) $$S\,e\,n\,s\,i\,t\,i\,v\,i\,t\,y=S\,n=\frac{T\,P}{T\,P+F\,N}$$

(15) $$S\,p\,e\,c\,i\,f\,i\,c\,i\,t\,y=S\,p=\frac{T\,N}{T\,N+F\,P}$$

(16) $$A\,c\,c\,u\,r\,a\,c\,y=A\,c\,c=\frac{T\,P+T\,N}{T\,P+T\,N+F\,P+F\,N}$$

(17) $$M\,C\,C=\frac{T\,P\times T\,N-F\,P\times F\,N}{\sqrt{\left(T\,P\times F\,P\right)\,\left(T\,P+F\,N\right)\,\left(T\,N+F\,P\right)\,\left(T\,N+F\,N\right)}}$$

where TP and TN are the numbers of correctly predicted positive and negative samples, respectively, as well as FP and FN are the numbers of wrongly predicted positive and negative samples, respectively. In addition, we also employed the receiver operating characteristic (ROC) to evaluate performances. The area under the ROC curve (AUC) lies between 0 and 1. The more the AUC, the better the performance.

## Results

There are two state-of-the-art methods for predicting TFs. One is the deep learning-based method by Li et al. (2022), which is called Li’s method, and another is the sequence feature-based method by Liu et al. (2020), which is called Liu’s method. To examine the Capsule_TF for efficiency and effectiveness in identifying TFs, we compared it with these two methods by the independent test. As shown in Table 1, the Capsule_TF is completely superior to the two methods. The Capsule_TF increased the Sn by 0.0283 over Li’s and even 0.1132 over Liu’s. The Capsule_TF increased MCC by 0.0386 over Li’s and even 0.1044 over Liu’s.

**TABLE 1:** Comparison with two states of the art methods in the independent test.

Method	Sn	Sp	Acc	MCC	AUC
Capsule_TF	**0.9151**	**0.8490**	**0.8820**	**0.7658**	**0.9252**
Li et al. (2022)	0.8868	0.8396	0.8663	0.7272	0.9130
Liu et al. (2020)	0.8019	0.8585	0.8302	0.6614	0.9116

The bold highlighted the best values.

## Discussion

### Effect of position

The length of amino acid sequences varies with TFs. The longest reached 4,834 amino acid residues, the shortest is only 51 residues, and each TFs have an average of 536 residues. It is compulsory that the input is of the unified length in the machine learning algorithm. We investigated the effects of the number of amino acid residues at different positions on discriminating TFs from non-TFs. We chose 500 amino acid residues at the start, at the middle, and the end, respectively. As shown in Table 2, their predictive performances are approximately equivalent, meaning that positions have little effect. A potential reason is that 500 amino acid residues might contain sufficient information about TFs.

**TABLE 2:** Predictive performance of amino acid residues from different positions.

Data	Sn	Sp	Acc	MCC	AUC
Upstream_500	0.9151	0.8490	0.8820	0.7658	0.9252
Centre_500	0.8773	0.8679	0.8726	0.7453	0.9084
Downstream_500	0.9056	0.8396	0.8726	0.7469	0.9149

### Contribution of capsule network

In comparison with Li’s method, the remarkable characteristic of the Capsule_TF is to utilize the capsule network. In order to investigate the contribution of the capsule network to classifying TFs, we removed it. The predictive performance after excluding the capsule network is listed in Table 3. Obviously, all metrics except Sp. decreased precipitously. Sn decreased from 0.9151 to 0.6320, Acc from 0.8820 to 0.7594, MCC from 0.7658 to 0.5365, and AUC from 0.9252 to 0.8120. The results indicated that the capsule network contributed much to identifying TFs.

**TABLE 3:** Predictive performance of the method without capsule network.

Method	Sn	Sp	Acc	MCC	AUC
Non-Capsule	0.6320	0.8867	0.7594	0.5365	0.8120
With-Capsule	0.9151	0.8490	0.8820	0.7658	0.9252

### Comparison with feature-based methods

**FIGURE 4:**
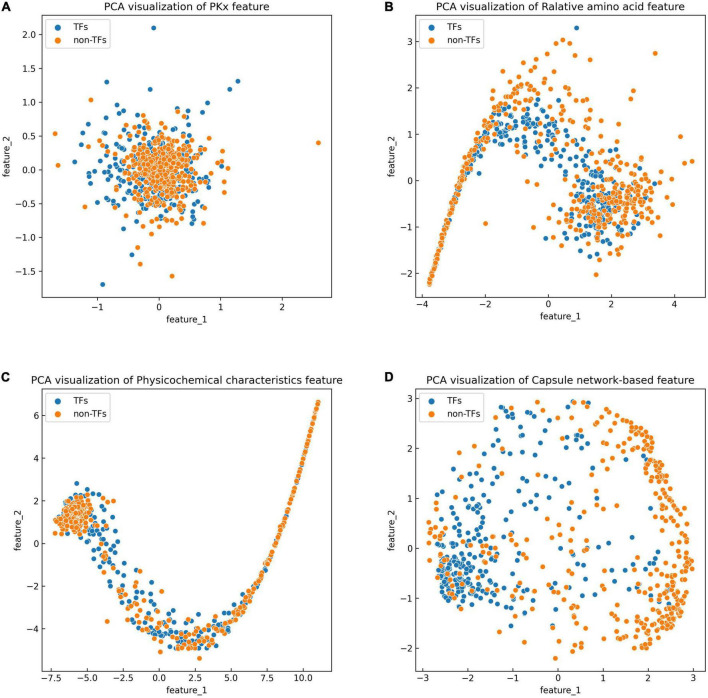
Principal component analysis (PCA) visualization about the different features: **(A)** PKx, **(B)** relative amino acid propensity, **(C)** physicochemical characteristics, and **(D)** capsule network-based features. As seen in this image, orange represents non-TFs and blue represents TFs.

The discriminative features provide a potential explanation to distinguish between both classes of samples. We compared three frequently used property-based features with the capsule network-based features. Three property-based features are PKx, relative amino acid propensity (RAA), and physicochemical characteristics (Li et al., 2008, 2021; Zhang et al., 2019). The output of the capsule layer was considered as the capsule network-based feature. Figure 4 visualizes the first two components of four types of features. The first two components were computed by PCA (Yang et al., 2004). Obviously, the first two components of the capsule network-based features are more discriminative than those of the other three types of features. We used the SVM (Noble, 2006) to compare the discriminative abilities of these features. As shown in Table 4, the capsule network-based feature is superior to the three property-based features. We also compared the logistic regression and LDA with the Capsule_TF. As listed in Tables 5, 6, the Capsule_TF is superior to the logistic regression and the LDA, and the capsule network-based features are superior to the conventional representations.

**TABLE 4:** Performance comparison across different features by SVM.

Feature	Sn	Sp	Acc	MCC
PKx	0.5660	0.7452	0.6556	0.3164
Relative amino acid propensity	0.6792	0.7075	0.6933	0.3869
Physicochemical characteristics	0.5283	0.6981	0.6132	0.2297
Capsule network-based feature	**0.9151**	**0.8396**	**0.8773**	**0.7568**

The bold highlighted the best values.

**TABLE 5:** Performance comparison across different features by logistic regression.

Feature	Sn	Sp	Acc	MCC
Pkx	0.7075	0.5660	0.6368	0.2764
Relative amino acid propensity	0.5943	0.6509	0.6226	0.2457
Physicochemical characteristics	0.6981	0.5849	0.6415	0.2849
Capsule network-based feature	0.9245	0.7924	0.8584	0.7233

**TABLE 6:** Performance comparison across different features by linear discriminant analysis (LDA).

	Sn	Sp	Acc	MCC
Pkx	0.6981	0.5094	0.6038	0.2113
Relative amino acid propensity	0.6321	0.5472	0.5896	0.1799
Physicochemical characteristics	0.7736	0.5189	0.6462	0.3024
Capsule network-based feature	0.8962	0.7830	0.8396	0.6836

The previous results indicated that the Capsule_TF outperformed two state-of-the-art methods: Li’s method (Li et al., 2022) and Liu’s method (Liu et al., 2020). Li’s method (Li et al., 2022) is a Bi-LSTM-based method, while Capsule_TF not only employed Bi-LSTM but also utilized a capsule network. The inclusion of a capsule network effectively promoted the representation of protein sequences of TFs. The ablation experiments validated the contribution of the capsule network to the identification of TFs (Table 3). Liu’s method (Liu et al., 2020) is feature-based. We compared features extracted by Capsule_TF with traditional sequence property-based features. As shown in Figure 4 and Table 4, the capsule network-based feature is more discriminative than the traditional sequence property-based feature. Despite the Capsule_TF obtaining superior performances over the state-of-the-art methods, there were some limitations that need to be improved in the feature. First, the consumption time in dynamic routing is very large. Therefore, Capsule_TF is not suitable to deal with large-scale datasets. Second, the interpretability of Capsule_TF needs to be improved.

### Web application

We realized the presented method into a web application which is freely available.^2^ The web application is based on the Django framework and utilized python and Tensorflow. The web application is very easy for users to use. The first thing is for the user to upload the predicted protein sequences in the FASTA format to the textbox or the file to the web. Clicking the “submit” button, users will obtain the results. The consuming time is directly proportional to the number of protein sequences. In addition, users could download the training and testing dataset in the experiments.

## Conclusion

The TFs are very influential in transcription regulation. It is a challenging task to accurately recognize TFs at present. We presented a capsule network-based method for identifying TFs, which outperformed the state-of-the-art methods in the experiments. The presented method benefits from the inclusion of a capsule network, which captures a more informative representation than the property-based method. We also developed a web application that facilitated the detection of TFs. The method and the web application are helpful to identify TFs and to further explore their roles. The TFs play typically regulating roles in gene expression by binding to short DNA sequences. The roles of TFs depend on their binding to DNA sequences. In the future, we hope to create an effective and efficient method to recognize such binding and interpret its mechanism from the semantics of both protein and DNA sequences.

## Data availability statement

The datasets presented in this study can be found in online repositories at http://www.biolscience.cn/Capsule_TF/.

## Author contributions

PZ: data curation, methodology, software, investigation, and writing. YQ: validation. XL, YL, and YY: conceptualization and writing. GH: conceptualization, funding acquisition, supervision, and writing – reviewing and editing. All authors contributed to the article and approved the submitted version.

## Footnotes

1
https://bioinfor.nefu.edu.cn/TFPM/

2
http://www.biolscience.cn/Capsule_TF/
